# Orthorexic tendencies in the general population: association with demographic data, psychiatric symptoms, and utilization of mental health services

**DOI:** 10.1007/s40519-020-00961-0

**Published:** 2020-07-29

**Authors:** Martin Greetfeld, Johannes Baltasar Hessler-Kaufmann, Beate Brandl, Tomas Skurk, Christina Holzapfel, Norbert Quadflieg, Sandra Schlegl, Hans Hauner, Ulrich Voderholzer

**Affiliations:** 1 Schoen Clinic Roseneck, Am Roseneck 6, 83209 Prien Am Chiemsee, Germany; 2 Department of Psychiatry and Psychotherapy, University Hospital, LMU Munich, Munich, Germany; 3 ZIEL–Institute for Food & Health, Technical University of Munich, Freising, Germany; 4 Chair of Nutritional Medicine, Else Kröner-Fresenius Center for Nutritional Medicine, Technical University of Munich, Freising-Weihenstephan, Germany; 5 Institute for Nutritional Medicine, School of Medicine, Technical University of Munich, Munich, Germany; 6 Department of Psychiatry and Psychotherapy, University Hospital of Freiburg, Freiburg, Germany

**Keywords:** Eating behaviors, Eating disorder, Mental health care, Population studies

## Abstract

**Purpose:**

Orthorexia nervosa (ON) is characterized by a preoccupation to eat healthily and restrictive eating habits despite negative psychosocial and physical consequences. As a relatively new construct, its prevalence and correlates in the general population and the associated utilization of mental health services are unclear.

**Methods:**

Adults from the general population completed the Düsseldorf Orthorexia Scale (DOS), the Patient Health Questionnaire (PHQ), the Short Eating Disorder Examination (SEED).

**Results:**

Five-hundred eleven (63.4% female) participants with a mean age of 43.39 (*SD *= 18.06) completed the questionnaires. The prevalence of ON according to the DOS was 2.3%. Considering only effects of at least intermediate size, independent samples *t*-tests suggested higher DOS scores for persons with bulimia nervosa (*p *< .001, Cohen’s *d *= 1.14), somatoform syndrome (*p *= .012, *d *= .60), and major depressive syndrome (compared *p *< .001, *d *= 1.78) according to PHQ as well as those who reported to always experience fear of gaining weight (*p *< .001, *d *= 1.78). The DOS score correlated moderately strong and positively with the PHQ depression (*r *= .37, *p *< .001) and stress (*r *= .33, *p *< .001) scores as well as the SEED bulimia score (*r *= .32, *p *< .001). In multivariate logistic regression analyses, only PHQ depression scores were associated with past psychotherapeutic or psychiatric treatment (OR = 1.20, *p *= .002) and intake of psychotropic medication in the last year (OR = 1.22, *p *= .013).

**Conclusions:**

The prevalence of ON was low compared to international studies but is in line with other non-representative German studies. Orthorexic tendencies related to general mental distress and eating disorder symptoms but were no independent reason for seeking treatment.

**Level of evidence:**

Level V, cross-sectional descriptive study.

While not being recognized as an official disorder, proposed criteria for orthorexia nervosa (ON) include a preoccupation with food and restrictive eating habits despite negative physiological, emotional, or psychosocial consequences [1]. Partly due to the questionable quality of the respective assessment tools [2–4] and the lack of a well-defined criterion for calculating optimal cut-offs, prevalence estimates of ON vary considerably. Despite limitations [3], the Düsseldorf Orthorexia Scale (DOS [5]) appears to be the most suitable questionnaire [4, 6] at the moment and has been translated to English [7], Spanish [8], and Chinese [9]. Hence, the following overview of the literature only includes studies using the DOS in the general population or student samples. Studies with selected populations like vegans [10] and athletes [11] were not considered, as these characteristics might influence prevalence rates and correlates of ON.

In a representative sample of the German population, the DOS with its proposed cut-off (above 95th percentile in the validation sample: score ≥ 30) yielded a prevalence of ON of 6.9% [12]. In non-representative German samples of varying size, including matched control groups of clinical samples and participants of an online survey, estimates range between 3 and 4% [5, 13–15]. These numbers are contrasted by studies from the USA [7], China [9], and Spain [16] that reported rates up to 10% in smaller student samples.

Higher DOS scores in general population samples are often associated with eating disorder symptoms [5, 9, 15, 16]. The higher rates in women suggested by some studies [5, 17, 18], however, are likely due to sample characteristics and not actual sex differences with regard to symptoms of ON [19]. The two studies that examined the association of DOS scores with psychiatric symptoms reported positive associations with depressive, anxious, obsessive, and compulsive symptoms, as well as negative associations with well-being and life satisfaction [12, 15]. One study reported higher rates of current psychotherapeutic treatment and use of psychotropic medication in persons with higher DOS scores [15]. Table 1 gives an overview of studies using the DOS for prevalence estimates and correlational analyses.

**Table 1:** Overview of studies employing the Düsseldorf Orthorexia Scale (DOS) in the general population

Study	Country	Sample			DOS total score		Correlates of higher DOS scores/ON
		Source	N (% female)	Age *M *± *SD*	% ON (above cutoff of ≥ 30)	*M *± *SD*	
Hennecke [17] in Barthels [18]	Germany	General population	2185 (n.a.)	n.a	1.7	n.a.	Female gender
Barthels [5]	Germany	General population	1340 (70)	29.3 ± 11.0	3	17.8 ± 5.4	Stronger drive for thinness, stronger bulimic symptoms, stronger body dissatisfaction, female gender
Depa [6]	Germany	Students	446 (70)	21.7 ± 2.6	3.3	18.4 ± 5.3	Lower semester
Barthels [13]	Germany	Control sample for patients with EDs from general population	33 (100)	23.2 ± 4.3	3.2	17.4 ± 4.6	n.a.
		Control sample for patients with OCD from general population	30 (40)	41.4 ± 13.3		15.9 ± 5.7	
Barthels [14]	Germany	Control sample for patients with EDs from general population	30 (100)	22.10 ± 7.43	n.a.	19.0 ± 4.5	n.a.
Barthels [10]	Germany	Control sample for dieters, vegans, and vegetarian	258 (77)	29.8 ± 11.0	1.5	16.6 ± 5.0	n.a.
Chard [7]	USA	Students	384 (70)	19.6 ± 2.6	8.0	20.0 ± 6.0	Vegetarianism, higher satisfaction with current diet
Strahler [15]	Germany	General population	713 (80)	29.4 ± 11.2	3.8	17.9 ± 5.5	Lower subjective social status, lower well-being, lower life satisfaction, more perceived stress, stronger restraint eating, stronger eating concern, stronger weight concern, stronger shape concern, stronger general eating pathology, stronger depressive symptoms, stronger anxiety symptoms, stronger obsessive symptoms, stronger compulsive symptoms, current psychotherapy, current psychotropic use
Luck-Sikorski [12]	Germany	Representative of general population	1007 (49)	50.6 ± .8	6.9	19.25 ± 6.11	Univariate: higher weight, lower education, vegetarianism, stronger depressive symptoms Multivariate: lower education, vegetarianism, stronger depressive symptoms
He [9]	China	Students	1075 (53)	20.1 ± 1.0	7.8	21.5 ± 5.4	Male gender, stronger eating inflexibility
Parra-Fernández [8]	Spain	Students	492 (57)	20.0 ± 3.0	10.5	17.52 ± 5.2	Stronger drive for thinness, stronger body dissatisfaction, stronger, perfectionism, lower interoceptive awareness, stronger asceticism

*M* mean, *SD* standard deviation, *n.a.* not available, *EDs* eating disorders, *ON* orthorexia nervosa as defined by a DOS score ≥ 30

Given the smaller number of prevalence estimates of ON based on the DOS in the general population and the limited data on the association of DOS scores with psychiatric symptoms as well as the utilization of mental health services, the aims of the present study were to examine (1) the prevalence of ON according to the DOS, (2) the association of DOS scores with demographic data and other psychiatric symptoms, as well as (3) the independent association of DOS scores with the utilization of psychotherapeutic and psychiatric treatment to explore to whether orthorexic tendencies are relevant for mental health services.

## Methods

### Participants and procedures

Participants (*N* = 511, 63.4% female) were recruited from two studies on nutrition and metabolism that were conducted at the Institute for Nutritional Medicine at the Technical University of Munich [20, 21]. Inclusion criteria for participation in these studies were age ≥ 18 years, body mass index (BMI) ≥ 18.5, absence of severe diseases, no surgery within the last 3 months, and no acute physical impairment. Using the existing personal data from these studies, the participants were contacted and asked whether they would like to take part in another study related to eating attitudes and habits. A set of questionnaires was completed either at the study center or mailed to the participants. A reminder was sent to those who did not respond after 1 month.

Mean age of the 511 participants was 43.4 years (SD = 18.1, range 18–84) and mean BMI was 25.2 kg/m2 (SD = 4.7, range 17.6–51.2). Nine participants (1.8%) had completed lower school education [German: Hauptschule], 37 (7.2%) had completed middle school education [German: Realschule], 67 (13.1%) had completed higher school education [German: Gymnasium], 134 (26.2%) had completed vocational training, and 262 (51.3%) had a university degree (data missing for 2 participants, .4%). All participants gave written informed consent. The study was approved by the institutional review boards of the University of Munich (#17-544) and the Technical University of Munich (#492/17S).

### Measures

The set of questionnaires included items on demographic data, current (at the time of questionnaire completion) and highest adult weight, current height, eating preferences (e.g., vegetarianism, veganism), current and past psychotherapeutic treatment, as well as current use of psychotropic medication. Further, the questionnaires included several validated scales.

Symptoms of ON were measured with the DOS [5]. Its 10 items inquire orthorexic eating behaviors (e.g., “I have certain nutrition rules that I adhere to”) and associated emotions (e.g., “If I eat something I consider unhealthy, I feel really bad”) and are rated on a four-point scale ranging from “this does not apply to me” (1) to “this applies to me (4). Total scores range from 0 to 40 and values between 25 and 29 represent risk of ON, while values ≥ 30 are considered to represent ON.

The German version of the Patient Health Questionnaire (PHQ [22]) assesses symptoms related to somatic symptom disorders, depressive disorders, anxiety disorders, eating disorders, alcohol misuse, and psychosocial functioning. The items allow for calculating sum scores (depression, somatic symptoms, and general stress) and categorical variables representing syndromes (depression, somatic symptoms, panic, bulimia nervosa, anorexia nervosa, alcohol misuse).

The Short Evaluation of Eating Disorders (SEED; [23]) is a German screening for eating disorder symptoms, which allows for the calculation of total severity indices for anorexia nervosa and bulimia nervosa, respectively. Its items ask for current height and weight, fear of gaining weight, body perception, and inappropriate compensatory behaviors. The latter three items are scored on five-point scales.

BMI and weight suppression (highest adult weight in kg–current weight in kg) were calculated as indices for current and past weight.

### Statistical analyses

The prevalence of ON is reported as the percentage of persons scoring 30 or above on the DOS. As a very small number of persons scored above the cut-off, further analyses used the DOS total score as continuous measure of orthorexic tendencies. Subsequent statistical associations are, hence, not to be understood as phenomena related to a categorical representation of a clinical syndrome of ON, but rather to higher or lower scores on the DOS.

Independent samples *t*-tests and univariate analyses of variance (ANOVAs) were conducted to compare mean DOS scores between levels of categorical variables. Statistically significant effects in the ANOVAs were followed-up with post hoc independent samples *t*-tests with Bonferroni-correction of the level of significance. Cohen’s *d* and partial η^2^ were calculated as measures of effect size for the *t* tests and the ANOVAs, respectively.

Associations of the DOS scores with continuous variables were examined with Pearson’s *r* correlation coefficients.

The adjusted association of DOS scores with the utilization of mental health care services was examined by three separate binary logistic regression analyses, with the use of the respective service (current psychotherapy, past psychotherapy, current use of psychotropic medication) as dichotomous (yes vs. no) dependent variable and the DOS score, the PHQ-D sum scores for depression, somatic symptoms, and stress, as well as the SEED anorexia and bulimia nervosa severity scales as continuous regressors.

Statistical analyses were performed with SPSS 25 for Macintosh. The two-tailed level of significance was set at .05. The interpretation of results was based on effect sizes following conventional recommendations [24, 25].

## Results

The mean DOS total score of the 511 participants was 16.47 (*SD* = 4.86, range 10–34). According to classifications recommended for the DOS, 474 (92.8%) had no ON, 25 (4.9%) were at risk, and 12 (2.3%) were supposed to have ON. The number of participants’ included in the following analyses may vary due to missing data.

Table 2 displays the frequency of the categorical variables, the corresponding mean DOS scores and the tests statistics for the mean comparisons. Independent *t* tests revealed statistically significantly higher mean DOS scores for women compared to men, persons with past psychiatric or psychotherapeutic treatment, current psychotherapeutic treatment, and psychotropic medication during the last year, as well as bulimia nervosa, somatoform syndrome, and alcohol syndrome according to the PHQ. Most effects, however, were of small to intermediate size. Large effect sizes were found for differences in DOS scores between persons with and without bulimia nervosa according to PHQ.

**Table 2:** Association of orthorexia nervosa symptom total score as measured with the Düsseldorf Orthorexia Scale (DOS) with categorical variables. Results of the univariate analyses of variance

Predictor	*N*	*M (SD*) DOS	Test statistics			Effect size
Sex^a^			*t *= 2.38 (467^c^), *p* = .018			*d* = .20 (small)
Female	324	16.83 (5.24)				
Male	187	15.84 (4.06)				
Highest education^b^			*F* = .15 (3, 505), *p* = .932			partial η^2^ = .001 (none)
Secondary school	46	16.30 (4.97)				
High school	67	16.57 (5.18)				
Occupational training	134	16.69 (4.96)				
University	262	16.39 (4.74)				
Past psychiatric or psychotherapeutic treatment^a^			*t *= 4.88 (508), *p* < .001			*d* = .49 (small)
Yes	135	18.19 (4.87)				
No	375	15.86 (4.61)				
Current psychiatric or psychotherapeutic treatment^a^			*t *= 3.52 (509), *p* < .001			*d* = .59 (intermediate)
Yes	39	19.08 (5.32)				
No	472	16.26 (4.76)				
Psychotropic medication during last year^a^			*t *= 4.09 (509), *p* < .001			*d* = .65 (intermediate)
Yes	44	19.30 (5.74)				
No	467	16.21 (4.69)				
Fear of gaining weight (SEED item 3)^b^			*F* = 26.71 (4, 505), *p* < .001			partial η^2^ = .174 (large)
Never	62	15.06 (3.96)				
Seldom	93	14.67 (3.55)				
Sometimes	161	15.54 (4.26)				
Often	122	17.11 (4.60)				
Always	72	21.02 (4.87)				
PHQ bulimia nervosa^a^			*t *= 5.01 (39.99^c^), *p* < .001			*d* = 1.14 (large)
Yes	38	21.38 (6.40)				
No	473	16.08 (4.50)				
PHQ somatoform syndrome^a^			*t *= 2.64 (41.09^c^), *p* = .012			*d* = .60 (intermediate)
Yes	39	19.12 (6.65)				
No	472	16.25 (4.63)				
PHQ depressive syndromes^b^			*F* = 27.52 (2, 509), *p* < .001			partial η^2^ = .098 (intermediate)
Major depressive syndrome	16	24.09 (6.27)				
Other depressive syndrome	62	17.98 (5.09)				
None	433	15.97 (4.49)				
PHQ alcohol syndrome^a^			*t *= .82 (509), *p* = .415			*d* = .14 (none)
Yes	40	17.08 (5.44)				
No	471	16.42 (4.81)				

*M* mean, *SD* standard deviation, SEED short evaluation of eating disorders, PHQ Patient Health Questionnaire

^a^Comparison of means with independent *t* test

^b^Comparison of means with univariate analysis of variance

^c^Degrees of freedom corrected due to unequal variances

Univariate ANOVAs revealed differences in mean DOS scores for different levels of fear of weight gain according to the SEED and depressive syndrome according to PHQ. Table 3 displays the results of the respective post hoc independent *t* tests. With regard to fear of weight, large effect sizes were found for the comparisons with the group of persons who reported to always experience this fear with them scoring higher on the DOS. Higher DOS sores with large effect sizes for the difference were found when comparing persons with major depressive syndrome with those with no or other depressive syndrome.

**Table 3:** Results of the post hoc independent *t*-tests for the univariate analyses of variance

Variable	*t* (*df*)	*p*	Cohen’s *d* effect size
Fear of gaining weight (SEED item 3)^a^			
Never vs. seldom	.65 (153)	.515	.11 (none)
Never vs. sometimes	.76 (221)	.447	.12 (none)
Never vs. often	2.98 (182)	.003	.47 (small)
Never vs. always	7.03 (125.97)	< .001	1.19 (large)
Seldom vs. sometimes	1.67 (252)	.096	.22 (small)
Seldom vs. often	4.39 (212.96)	< .001	.58 (intermediate)
Seldom vs. always	8.21 (111.18)	< .001	1.36 (very large)
Sometimes vs. often	2.96 (281)	.003	.36 (small)
Sometimes vs. always	7.22 (106.89)	< .001	1.15 (large)
Often vs. always	4.90 (123.75)	< .001	.77 (intermediate)
PHQ depressive syndromes^b^			
None vs. other depressive syndrome	3.24 (493)	.001	.44 (small)
None vs. major depressive syndrome	5.13 (15.57)	< .001	1.78 (large)
Other depressive syndrome vs. major depressive syndrome	4.08 (76)	< .001	1.14 (large)

^a^Bonferroni-corrected level of significance .05/10 = .005

^b^Bonferroni-corrected level of significance .05/3 = .017

^c^Degrees of freedom corrected due to unequal variances

Table 4 displays the results for the associations of the DOS total score with continuous variables. Higher DOS total scores were statistically significantly associated with lower age, higher adult lifetime BMI, higher weight suppression, higher PHQ scores for depression, somatoform symptoms, and stress, as well as higher SEED scores for anorexia nervosa and bulimia nervosa. Yet, effect sizes were small or intermediately large.

**Table 4:** Association of Düsseldorf Orthorexia Scale (DOS) scores ›with continuous variables. Results of the bivariate correlation analyses

Predictor	*N*	Pearson *r* (effect size)	*p*
Age	503	− .15 (small)	.001
Current BMI	507	.06 (none)	.158
Highest adult lifetime BMI	508	.17 (small)	< .001
Weight suppression	505	.23 (small)	< .001
PHQ depression score	501	.37 (intermediate)	< .001
PHQ somatoform score	414	.29 (small)	< .001
PHQ stress score	504	.32 (intermediate)	< .001
SEED anorexia nervosa score	473	.29 (small)	.004
SEED bulimia nervosa score	505	.32 (intermediate)	< .001

*BMI* body mass index, *SEED* Short Evaluation of Eating Disorders, *PHQ* Patient Health Questionnaire

Table 5 displays the results of the multiple binary logistic regression analyses for mapping the independent association of DOS scores with use of the mental health care system. Adjusted for depressive symptoms, stress, anorexia nervosa symptoms, bulimia nervosa symptoms, age, and sex, the DOS score was not associated with past psychiatric or psychotherapeutic treatment, nor current psychiatric or psychotherapeutic treatment, nor use of psychotropic medication within the last year. Only depressive symptoms and age showed statistically significant associations.

**Table 5:** Adjusted association of DOS total scores with utilization of mental health care services. Results of the binary logistic regression analyses

Model	Odds ratio	95% CI	*p*
1: Past psychiatric/psychotherapeutic treatment: Nagelkerke’s R^2^ = .22			
PHQ depression score	1.21	1.08–1.36	.002
PHQ stress score	.95	.86–1.15	.945
SEED anorexia nervosa score	1.08	.36–3.27	.894
SEED bulimia nervosa score	1.60	.84–3.03	.152
Age in years	1.02	1.00–1.03	.039
Male gender	.62	.36–1.06	.079
DOS total score	1.04	.99–1.10	.103
2: Current psychiatric/psychotherapeutic treatment: Nagelkerke’s R^2^ = .23			
PHQ depression score	1.11	.95–1.30	.190
PHQ stress score	1.16	.95–1.43	.149
SEED anorexia nervosa score	.82	.11–6.35	.849
SEED bulimia nervosa score	2.04	.80–5.17	.135
Age in years	1.00	.98–1.03	.772
Male gender	.41	.14–1.19	.101
DOS total score	1.00	.92–1.08	.983
3: psychotropic medication during last year: Nagelkerke’s R^2^ = .20			
PHQ depression score	1.22	1.04–1.43	.013
PHQ stress score	1.00	.82–1.22	.996
SEED anorexia nervosa score	1.76	.26–12.16	.564
SEED bulimia nervosa score	.93	.37–2.35	.885
Age in years	1.02	1.00–1.04	.106
Male gender	.58	.23–1.45	.244
DOS total score	1.05	.98–1.13	.198

*95% CI* 95% confidence interval of the odds ratio, *SEED* short evaluation of eating disorders, *PHQ* patient health questionnaire, *DOS* Düsseldorf Orthorexia Scale

## Discussion

With regard to our study aims we found: (1) The prevalence of ON according to the DOS was 2.3%, with another 4.9% being at risk of developing ON. Hence, the overall load of orthorexic tendencies in the sample was low and “higher DOS scores” are to be understood as relatively higher but not near or above a cut-off. (2) Only considering effects with at least intermediate size, higher mean DOS score were found for persons, who reported to always experience a fear of gaining weight and who met the classifications of bulimia nervosa, somatoform syndrome, or major depressive syndrome according to the PHQ. Correlational analyses suggested higher DOS scores relating to higher PHQ depression and stress scores, as well as higher SEED bulimia nervosa scores. (3) While persons with past or current psychiatric treatment also showed higher DOS scores in univariate analyses, these effects vanished in multivariate logistic regression analyses. The probability of using or having used mental health services only increased with higher depressive symptoms according to PHQ and higher age.

With values around 1.5%, only two studies [10, 17] reported lower prevalence estimates for ON based on the DOS than we found, with the study by Hennecke being more than 10 years old and employing a preliminary version of the DOS. Among the studies with the DOS, there seems to be no clear pattern connecting sample characteristics, such as sample size, age, or percentage of female participants with the prevalence of ON. It is striking, however, that in non-representative samples from the German general population, estimates lie around 3% [5, 6, 10, 13, 15, 17], while the only representative German study [12] and all non-German studies yield values ranging from 7 to 10% [7, 9, 16]. While differences might be attributable to the younger mean age of the student samples used for validating the Chinese, Spanish, and English versions of the DOS, the high estimate of the representative German sample comes as a surprise. Luck-Sikorski et al. [12] argue that the high prevalence in their sample might result from an increased incidence of ON over the years. The more recent and lower prevalence estimates in our and another German study [15] contradict this explanation and suggest that variables inherent to the samples or study designs explain the discrepancies.

It is still debated whether ON represents a circumscribed clinical entity and whether this entity should be allocated to the eating disorders [1, 26]. Orthorexic tendencies are strongly associated with core eating disorder symptoms like body dissatisfaction and drive for thinness [5, 15, 16, 27], which was further confirmed by our data. Together with a high prevalence of ON in anorexia nervosa [14], this finding cannot easily be reconciled with the proposition that restrictive eating in ON is not aimed at weight loss but at healthy eating [1]. However, there was no association between orthorexic tendencies and measures of past and present weight in our sample, unlike to what is known from eating disorders [28, 29]. Also, the lack of an association between orthorexic tendencies and symptoms of anorexia nervosa in our sample does not concur with what is reported for clinical and non-clinical samples (see Table 1; [13, 14]). There was, however, tentative evidence in our sample for an association between orthorexic tendencies and symptoms of bulimia nervosa, which corresponds with previous studies (see Table 1, e.g., [5]) and suggests a relationship between orthorexic tendencies and impulsive rather than restrictive eating. The counterintuitive nature of this association warrants further examination.

The association with other psychiatric symptoms, especially those of depression, concur with preliminary results [12]. Even though two studies using the DOS reported conflicting results with regard to sex differences [9, 17], the small effect size for differences in DOS scores between men and women in our study confirms the general literature, which suggests no association of measures of ON with sex [19]. The notion that ON is more prevalent in lower educational levels [12] was not supported by our data.

The only study examining the use of mental health care in relationship to ON found higher rates of current psychotherapy and use of psychotropic medication to be associated with higher ON scores [15]. Our univariate analyses replicated these relationships and extended them by indicating that persons with higher DOS scores were more likely to having received psychotherapy or psychiatric treatment in the past. When adjusting for other psychiatric symptoms, however, these associations vanished and depressive symptoms emerged as independent predictor together with higher age. These results suggest that orthorexic tendencies alone might not be a primary reason for seeking treatment.

Overall, it seems as orthorexic tendencies are related to general mental distress and some symptoms that are typical for eating disorders. Given the high comorbidity between eating disorders and depressive and other mental disorders [30, 31], this finding is compatible with the notion that ON in its clinical form may rather be some form of eating disorder. This, however, does not contradict the conceptualization of ON being exclusively aimed at healthy eating.

Our study is limited by the fact that the sample was derived from the general population, yet, drawn from studies on health and nutrition, which may have introduced bias into the sample through including a substantial proportion of persons with either specific interests in nutrition or related health problems. The low prevalence of ON that is in line with several other studies from the general population, however, contradicts this notion. Further, we had no means to assess any difference between those who returned the questionnaire and those who did not.

## Conclusions

Given the relatively low prevalence of ON in our study and other samples, the symptomatic overlap with established eating disorders, and the fact that stronger ON symptoms alone do not seem to be a reason for consulting mental health care professionals, there is little indication to view ON as an independent public health issue. Rather, ON may be an eating style overlapping with eating disorders and a maladaptive coping mechanism [14] in the face of general stress, including growing public pressure to eat healthy, and depression.

## What is already known on this subject?

Orthorexic symptoms show varying prevalence rates in the general population depending on instrument and sample characteristics, as well as unspecific associations with measures of psychiatric symptoms.

## What this study adds?

The study substantiates previous prevalence estimates and emphasizes that orthorexic tendencies relate to general mental distress, but are no independent reason for utilizing health care services.
